# Evaluation of three polygenic risk score models for the prediction of breast cancer risk in Singapore Chinese

**DOI:** 10.18632/oncotarget.24374

**Published:** 2018-01-31

**Authors:** Claire Hian Tzer Chan, Prabhakaran Munusamy, Sau Yeen Loke, Geok Ling Koh, Audrey Zhi Yi Yang, Hai Yang Law, Chui Sheun Yoon, Chow Yin Wong, Wei Sean Yong, Nan Soon Wong, Raymond Chee Hui Ng, Kong Wee Ong, Preetha Madhukumar, Chung Lie Oey, Gay Hui Ho, Puay Hoon Tan, Min Han Tan, Peter Ang, Yoon Sim Yap, Ann Siew Gek Lee

**Affiliations:** ^1^ Division of Medical Sciences, Humphrey Oei Institute of Cancer Research, National Cancer Centre, Singapore; ^2^ DNA Diagnostic and Research Laboratory, KK Women's and Children's Hospital, Singapore; ^3^ Department of General Surgery, Singapore General Hospital, Singapore; ^4^ Department of Surgical Oncology, National Cancer Centre, Singapore; ^5^ Department of Medical Oncology, National Cancer Centre, Singapore; ^6^ Oncocare Cancer Centre, Gleneagles Medical Centre, Singapore; ^7^ Koong and Ho Surgery Centre, Singapore; ^8^ Department of Pathology, Singapore General Hospital, Singapore; ^9^ Institute of Bioengineering and Nanotechnology, Singapore; ^10^ Lucence Diagnostics Pte Ltd, Singapore; ^11^ Department of Physiology, Yong Loo Lin School of Medicine, National University of Singapore, Singapore; ^12^ Office of Clinical and Academic Faculty Affairs, Duke-NUS Graduate Medical School, Singapore

**Keywords:** breast cancer, single-nucleotide polymorphism, risk loci, genotyping, polygenic risk score

## Abstract

Genome-wide association studies (GWAS) have proven highly successful in identifying single nucleotide polymorphisms (SNPs) associated with breast cancer (BC) risk. The majority of these studies are on European populations, with limited SNP association data in other populations. We genotyped 51 GWAS-identified SNPs in two independent cohorts of Singaporean Chinese. Cohort 1 comprised 1294 BC cases and 885 controls and was used to determine odds ratios (ORs); Cohort 2 had 301 BC cases and 243 controls for deriving polygenic risk scores (PRS). After age-adjustment, 11 SNPs were found to be significantly associated with BC risk. Five SNPs were present in <1% of Cohort 1 and were excluded from further PRS analysis. To assess the cumulative effect of the remaining 46 SNPs on BC risk, we generated three PRS models: Model-1 included 46 SNPs; Model-2 included 11 statistically significant SNPs; and Model-3 included the SNPs in Model-2 but excluded SNPs that were in strong linkage disequilibrium with the others. Across Models-1, -2 and -3, women in the highest PRS quartile had the greatest ORs of 1.894 (95% CI = 1.157–3.100), 2.013 (95% CI = 1.227–3.302) and 1.751 (95% CI = 1.073–2.856) respectively, suggesting a direct correlation between PRS and BC risk. Given the potential of PRS in BC risk stratification, our findings suggest the need to tailor the selection of SNPs to be included in an ethnic-specific PRS model.

## INTRODUCTION

Advances in technology and large collaborative efforts have led to the success of genome-wide association studies (GWAS) in their discovery of multiple breast cancer (BC)-associated risk loci. Researchers are now able to identify regions or genes that were not previously thought to be associated with BC risk. To date, over 100 single nucleotide polymorphisms (SNPs) have been identified. Though many of these SNPs were identified in predominantly Caucasian populations [1–8], there are a handful of SNPs identified in Asian populations as well [9–15]. Many groups have also attempted to replicate these associations in larger cohorts and/or in cohorts of different ethnicities. However, some SNPs have been shown to be ethnic-specific and do not necessarily replicate in other ethnicities [12, 14, 16–23]. Fine-scale mapping has subsequently been carried out to identify functional SNPs associated with BC risk in a particular ethnic group [16, 23]. In more recent years, fine-scale mapping of regions identified by GWAS [24–27] and meta-analysis of existing GWAS [28–33] have also contributed to the growing number of SNPs associated with BC susceptibility. As breast cancer is a highly heterogeneous disease, association studies have also been performed to discover risk loci specific to a particular breast cancer histological type or hormone receptor subtype [3, 4, 8, 17, 28, 30, 33–36].

Though it has been demonstrated that these SNPs are associated with BC risk, the risk that a single variant confers is relatively low. Several groups have attempted to generate polygenic risk scores (PRS) derived from a combination of different selected SNPs to evaluate the cumulative effect of these SNPs [37, 38]. A PRS considers the odds ratio (OR) of each SNP and the total number of risk alleles an individual carries.

As new risk loci have recently been discovered [26, 27, 32, 33], this current study aimed to assess the association of these SNPs with BC risk in Singapore Chinese. Well-established BC risk-associated SNPs as well as 13 recently discovered SNPs that have not been previously genotyped in Asian populations were evaluated to determine if these SNPs are associated with BC risk in our Singapore Chinese population, and combinations of SNPs were used to generate PRS.

## RESULTS

### Genotyping and association of SNPs with BC risk

Genotyping of the 51 SNPs (Supplementary Table 1) was carried out on 1,670 BC patients and 1,189 healthy controls of Chinese ethnicity. After excluding samples that failed to reach 95% call rate for all assays, samples were further separated into two independent cohorts; Cohort 1 included 1294 cases and 885 controls to determine the association of the SNPs with BC risk, and Cohort 2 included 301 cases and 243 controls to derive PRS models. The demographics and clinico-pathological characteristics of these cases and controls are summarized in Supplementary Table 2. The mean age of cases and controls in Cohort 1 was 50.2 years and 42.7 years respectively, and that of Cohort 2 was 49.9 years and 42.0 years respectively. The differences in age between cases and controls in both cohorts were statistically different (*P* < 2.2 × 10^–16^).

All SNP assays had a call rate of more than 95.0% with an average call rate of 99.1%, and did not deviate from Hardy-Weinberg Equilibrium in controls. Five SNPs, rs554219, rs614367, rs75915166, rs78540526, and rs56069439 were present in less than 1% of Cohort 1, and were excluded from further PRS analysis. Associations of the remaining 46 SNPs with BC risk in our Singapore Chinese cohort are reflected in Supplementary Table 3.

Results from logistic regression analysis with and without age-adjustment revealed 10 common SNPs to be statistically significant via an additive model at *P* < 0.05 (Supplementary Table 3). It was also observed that another SNP, rs2981579, which was found to be significant in the analysis without age-adjustment, was no longer significant after age-adjustment. An additional SNP, rs745570, was also found to be significantly associated with BC risk only after age-adjustment.

### Development of PRS models and their association with BC risk

PRS were generated based on unadjusted and age-adjusted ORs for 3 models: (1) Model-1 included all 46 SNPs investigated in this study; (2) Model-2 only included 11 statistically significant SNPs; and (3) Model-3 included 9 SNPs, after excluding SNPs in strong linkage disequilibrium (LD) with other SNPs (Supplementary Table 3). The PRS were identified to be statistically significant for BC risk, across all models (Table 1). It was also observed that across all models, the PRS ORs were higher for the 4th quartile when compared to the 1st quartile (Table 1). For instance, when using Model-1 which included unadjusted ORs of 46 SNPs, women in the 4th quartile had a 1.88-fold higher risk of BC compared to the 1st quartile. Similarly, with age-adjusted ORs the increase in BC risk was 1.89-fold.

**Table 1:** Association analysis with and without age-adjustment between breast cancer risk and polygenic risk score (PRS) for three PRS models

PRS quartile	Controls (*n*)	No. of SNPs included in the model	PRS derived from unadjusted ORs			PRS derived from age-adjusted ORs		
			Cases (n)	OR (95% CI)	*P*-value	Cases (*n*)	OR (95% CI)	*P*-value
**Model-1**								
1st	61	46	53	Ref	-	51	Ref	-
2nd	61		72	1.358 (0.823–2.244)	0.231	72	1.412 (0.852–2.338)	0.180
3rd	61		78	1.472 (0.895–2.421)	0.128	83	1.627 (0.990–2.677)	0.055
4th	60		98	1.880 (1.153–3.064)	0.011	95	1.894 (1.157–3.100)	0.011
Trend	243		301	1.325 (1.099 to 1.597)	0.003	301	1.301 (1.070–1.587)	0.009
**Model-2**								
1st	61	11	48	Ref	-	49	Ref	-
2nd	61		72	1.542 (0.928–2.562)	0.095	76	1.551 (0.936–2.570)	0.088
3rd	61		74	1.500 (0.901–2.496)	0.119	79	1.612 (0.975–2.666)	0.063
4th	60		107	2.266 (1.384–3.710)	0.001	97	2.013 (1.227–3.302)	0.006
Trend	243		301	1.312 (1.077–1.600)	0.007	301	1.267 (1.051–1.568)	0.0293
**Model-3**								
1st	61	9	54	Ref	-	54	Ref	-
2nd	61		72	1.333 (0.808–2.199)	0.260	72	1.333 (0.808–2.199)	0.260
3rd	61		77	1.426 (0.867–2.344)	0.162	82	1.519 (0.927–2.488)	0.097
4th	60		98	1.845 (1.134–3.003)	0.014	93	1.751 (1.073–2.856)	0.025
Trend	243		301	1.608 (1.149–2.250)	0.006	301	1.480 (1.039–2.120)	0.031

Finally, the AUC for each of the different PRS models were obtained to evaluate how effective each model was. Model-1 had the highest AUC value among the 3 PRS models for unadjusted and age-adjusted ORs of 0.572 (95% CI = 0.523–0.620) and 0.566 (95% CI = 0.517–0.614) respectively. AUCs of Model-2 and -3 using unadjusted ORs were 0.570 (95% CI = 0.522–0.619) and 0.566 (95% CI = 0.517–0.614) respectively, while that of Model-2 and -3 using age-adjusted ORs were 0.565 (95% CI = 0.516–0.613) and 0.557 (95% CI = 0.508–0.606) respectively.

## DISCUSSION

We assessed the association of 46 GWAS-identified SNPs with BC risk in Singapore Chinese and identified 11 SNPs to be significantly associated with increased BC risk. We also generated a PRS to measure the cumulative effect of variants, and to determine its discriminatory ability by means of AUC. Compared to other studies that have utilized PRS (Supplementary Table 4), this current study has included 7 new SNPs that have not been previously included in any other PRS. We have observed similar AUCs in our study as compared to previous studies, both in European and Asian populations (Table 2).

**Table 2:** Comparison of the studies on polygenic risk score (PRS) for breast cancer risk

Reference	Our study			Lecarpentier *et al.,* 2017 [44]			Hsieh *et al.,* 2017 [26]	Wen *et al.,* 2016 [7]	Mavaddat *et al.,* 2015 [6]	Vachon *et.al.,* 2015 [13]	Lee *et al.,* 2014 [25]	Zheng *et al.,* 2010 [12]
**Study population**	Singapore Chinese			Caucasian (Male BRCA1/2 mutation carriers)			Taiwanese	East Asians	European	Caucasian	Singapore Chinese	Chinese
**Cases**	**All**			**All**	**ER**^+‡^	**ER**^–‡^	446	11,760	33,673	1,643	411	3,039
	301			277	277	277						
**Controls**	243			1,469			514	11,612	33,381	2,397	1,212	3,082
**No. of SNPs studied** **(No. of SNPs included in PRS)**	**Model-1**	**Model-2**	**Model-3**	102 (88)	102 (87)	102 (53)	13 (6)	78 (44)	77 (77)	76 (76)	51 (51)	12 (8)
	51 (46)	51 (11)	51 (9)									
**AUC**	0.566	0.565	0.557	0.59	0.59	0.55	0.598	0.606	0.622	0.68	-	0.63

^‡^SNPs found to be associated with ER+ and ER- negative BC from other published literature were used to derive the ER+ and ER− specific PRS respectively.

There has not been a common consensus on whether fewer or a greater number of SNPs would render a better PRS model. In two separate studies conducted in Asians, one obtained an AUC of 0.63 using only 8 SNPs in their PRS [39] while the other obtained an AUC of 0.606 using a 44-SNP PRS [38]. Both Asian studies had evaluated an initial higher number of SNPs but only included SNPs that were found to be statistically significant in their own study cohort for the calculation of their PRS. In comparison, a European study had an AUC of 0.68 obtained from a PRS model which included 76 SNPs [40]. These findings suggest a need to tailor the selection of SNPs to be specific for the populations being studied.

In addition, due to the significant differences in age between cases and controls, we performed age-adjustment for the determination of ORs of SNPs and PRS. We observed similar trends of ORs and PRS for both unadjusted and age-adjusted analysis, suggesting that PRS as a predictor for BC risk is independent of age in our population.

Using age-adjusted ORs, we constructed a PRS using the 11 SNPs found to be significantly associated with BC risk (Model-2) and obtained an AUC of 0.565. As some SNPs were in LD with each other and may thus be over-represented, we constructed a 9-SNP PRS which only included the SNPs with the strongest association in each LD block (Model-3). However, Model-3 had a slightly weaker discriminatory ability with an AUC of 0.557 as compared to Model-2. By generating a PRS with all 46 SNPs studied, a similar AUC was observed at 0.566. Though the remaining 35 SNPs, including 11 out of the 12 SNPs recently discovered by Michailidou *et al*. [32], were not found to be statistically significant with BC risk in our study, it is possible that some of these SNPs failed to reach statistical significance as our study could have had insufficient power to detect the associations and additional studies of Asian ancestry are thus warranted to confirm if these SNPs are significantly associated with BC risk. GWAS and other discovery methods could also be done on Asian populations to further identify novel ethnic-specific SNPs that could have more significant associations with BC risk in Asians [41].

Of the 11 SNPs found to be statistically significant in our study, 4 SNPs were located on 6q25.1 (*ESR1*). 6q25.1 (*ESR1*) as a BC susceptible locus was first identified in Chinese [9], and additional SNPs in this region have been found to be associated with BC risk [6, 33, 42]. The SNPs with the strongest association with BC risk identified in our study (rs3757318, rs11155804, rs12662670 and rs2046210) were all located within this locus and each caused an increase in BC risk of about 40%, similar to previous studies carried out on Chinese [9] and South-East Asians [43]. It has been also observed that these variants tend to increase risk by a higher magnitude in Asians as compared to Europeans [42, 43], suggesting the importance of 6q25.1 as a BC susceptible region particularly in Asians. It is noted that the four SNPs exhibited the same statistical tendency and had similar ORs as they were in LD with each other.

Other significant associations identified in our study included variants on 5q11.2-*MAP3K1* (rs16886165), 9q31.2-*CHCHD4P2* (rs10816625), 10q22.3-*ZMIZ1* (rs704010), 11p15.5-*TNNT3* (rs909116), 12p11.2-*PTHLH* (rs7297051), 16q12.1-*TOX3* (rs4784227), and 17q25-*CBX8* (rs745570). With the exception of rs745570, all these other SNPs have been previously reported to be significantly associated with BC risk in Asian populations, with similar ORs and direction of effect. Rs745570 which maps to 17q25 (*CBX*8) was recently identified by Michailidou *et al.* [32]. Though a recent study has demonstrated that the expression of *CBX8* promotes mammary tumorigenesis both *in vivo* and *in vitro* [44], information on 17q25 (*CBX*8) as a breast cancer susceptibility locus is limited. To the best of our knowledge, our study is the first to validate and confirm the association of rs745570 with increased BC risk in an Asian population.

10q21.2 (*FGFR2*) was one of the first BC susceptibility locus to be identified by early GWAS [1, 2]. Rs11200014, rs1219648, rs2981579 and rs2981582 on 10q21.2 have been found to be associated with BC risk across different ethnicities, and the variant alleles tend to have a slightly greater effect in Europeans (ORs of 1.23 to 1.31) as compared to Asians (ORs of 1.15 to 1.23) [1, 2, 45–47]. Similarly, we observed lower ORs of 1.13 to 1.15 in Singapore Chinese. Though these associations were only found to be of borderline significance, we should not discount the importance of *FGFR2* as a BC susceptibility locus in our population.

In addition, our study is the first to investigate the associations of rs554219, rs75915166 and rs78540526, which map to 11q13.3 (*CCDN1*), with BC risk in Asians. We also included an additional SNP at the same locus, rs614367, which has one of the strongest associations with BC risk and was one of the first few risk loci identified by GWAS [6]. The association of rs614367 with BC risk has also been confirmed in Asians [18]. These four SNPs were initially removed from association analysis as they were found in less than 1% of our cohort. Likewise, an earlier study has also demonstrated that the variant alleles of these SNPs are much rarer in Asians as compared to Europeans [48]. Notably, the ORs for these four SNPs at *CCDN1* ranged from 2.64 to 4.87, and were higher than the other SNPs in this study. As these rare variants are present in low frequencies, sufficiently powered studies of greater sample sizes are needed to further validate these findings.

Though the discriminatory ability of a PRS model has been inadequate for clinical use, it has considerable potential in improving risk modeling. It has been demonstrated that PRS models aid in refining the risk stratification of individuals who are already at an increased risk of developing breast cancer [37, 40, 49, 50]. Some groups have attempted to combine PRS with other BC risk factors, such as breast density [40] or features included in the Gail Model [51], and improvements to AUCs have been observed. In a study by Shieh *et al.* [52], the addition of a BCSC (Breast Cancer Surveillance Consortium) risk score derived from information on age, ethnicity, first-degree relatives with BC, personal history of prior biopsies and breast density improved the AUC from 0.60 to 0.65. In a separate study by Hsieh *et al.* [53], other factors such as age of menarche and menopause, parity and body mass index were added to the PRS to improve the AUC from 0.598 to 0.665.

In summary, we have identified 11 SNPs out of the 46 SNPs that were significantly associated with BC risk in our Singapore Chinese cohort. We have also evaluated 3 different PRS models, with the model that included all 46 SNPs performing the best. In addition, we performed logistic regression analysis based on PRS quartiles which showed an overall trend across models and groups, and the highest quartile predicted to have the highest OR thus implying a direct correlation between PRS and OR. By improving risk prediction models, we will not only better stratify individuals according to their risk groups, but we could potentially also provide more efficient and effective screening and prevention methods.

## MATERIALS AND METHODS

### Study cohort

The study utilised DNA from 1,670 patients diagnosed with BC and 1,189 healthy controls with no known disease upon recruitment. All samples were obtained from women of Chinese ancestry. Peripheral blood samples were either obtained from unselected BC patients attending outpatient clinics at the National Cancer Centre and Singapore General Hospital or were archival frozen peripheral blood samples of BC patients from the SingHealth Tissue Repository. DNA was extracted using an optimized in-house method [54]. Control samples comprised of archival DNA acquired from the DNA Diagnostic and Research Laboratory, KK Women's and Children's Hospital, Singapore. Ethics approval for the study was given by the SingHealth Centralized Institutional Review Board (CIRB Ref: 2008/478/B), and written informed consent was taken from each participant.

### SNP selection

The association of 51 SNPs with BC susceptibility was assessed (Supplementary Table 1). SNPs were selected based on two criteria: (1) the SNPs were significantly associated with BC risk at a genome-wide level (*P* value = 5 × 10^–8^); (2) SNPs found to be monomorphic in Chinese were excluded. Well-established BC risk-associated SNPs [1, 2, 5–8, 15, 17, 29, 30, 39, 42, 46, 48, 55] were selected, as well as more recently identified SNPs [13, 26, 27, 56–59], including 12 from the recent study by Michailidou *et al.* [32].

### SNP genotyping

High-throughput genotyping for the 51 SNPs was carried out on 192.24 Dynamic Array^TM^ integrated fluidic circuits (IFC) (Fluidigm, CA, USA). TaqMan^®^ SNP Genotyping Assays (Applied Biosystems, CA, USA) were employed, and the BioMark HD (Fluidigm) was used for thermal cycling and fluorescence detection. Raw intensity data were converted to genotype calls based on k-means clustering using the Fluidigm SNP Genotyping Analysis software.

### Statistical analysis

#### SNP association analysis

A case-control study design was used to determine the association between the SNPs and BC. Cohort 1 comprised of 1294 cases and 885 controls, and only samples with a SNP genotype call rate of ≥95% were included. Using the PLINK tool [60], logistic regression analysis was carried out to identify statistically significant SNPs associated with BC. In addition, we performed logistic regression analysis using age as a covariate along with individual SNPs to determine its effect on BC risk and calculated the age-adjusted ORs along with its statistical significance. A *P*-value of ≤ 0.05 was considered statistically significant.

#### Linkage disequilibrium analysis

Using the PLINK toolset, LD analysis of the SNPs was performed to determine their non-random association in our population. The LD pattern between SNPs were measured using the correlation coefficient, *r^2^*, where *r^2^* ≥ 0.5 was considered moderate to strong.

#### Polygenic risk score analysis

An additional independent cohort with 301 cases and 243 controls (Cohort 2) was used to construct the PRS. We only considered SNPs with a minor allele frequency >1% within our cohort from the SNP risk association analysis to be included in the PRS models. To assess the cumulative effect of the SNPs, we calculated a PRS by summing the logOR of the SNP multiplied by the number of risk alleles of the SNP across all selected SNPs in an individual [37]. Two different PRS were calculated for overall BC risk; using unadjusted and age-adjusted ORs. Further, for each group, we derived three different PRS models based on varying numbers of SNPs to be included in the model. Model-1 included 46 SNPs found to be significantly associated with BC from published studies (Supplementary Table 1); Model-2 included statistically significant SNPs (*P*-value ≤ 0.05) associated with BC; Model-3 included statistically significant SNPs (*P*-value ≤ 0.05) but excluded SNPs that were in moderate to strong LD (*r^2^* ≥ 0.5) with each other.

To investigate the association between BC and PRS, logistic regression analysis was performed with PRS being a continuous variable [37]. In addition, ORs based on logistic regression models were estimated for different PRS quartiles with the first quartile being the reference. Finally, to determine the discriminating ability of the model, the area under the receiver operating characteristic (AUC) was estimated. Statistical analyses were performed using R version 3.4.1 and PASW statistics 18 software.

## SUPPLEMENTARY MATERIALS TABLES











## Abbreviations

AUC: area under the curve
BC: Breast Cancer
BCSC: Breast Cancer Surveillance Consortium
CI: confidence interval
GWAS: genome-wide association study
LD: linkage disequilibrium
OR: odds ratio
PRS: polygenic risk score
SNP: single nucleotide polymorphism
