# Supplementary material for: Evaluation of three polygenic risk score models for the prediction of breast cancer risk in Singapore Chinese
Source: Oncotarget. 2018 Jan 31;9(16):12796–804. doi: 10.18632/oncotarget.24374 (PMC5849174; doi:10.18632/oncotarget.24374)
Supplement: Supplementary file 1 [file oncotarget-09-12796-s001.pdf]

Evaluation of three polygenic risk score models for the prediction of breast cancer risk in Singapore Chinese

SUPPLEMENTARY MATERIALS

Supplementary Table 1: List of 51 SNPs. See Supplementary_Table_1

Supplementary Table 2: Participant Characteristics. See Supplementary_Table_2

Supplementary Table 3: Association of the 46 SNPs with BC risk. See Supplementary_Table_3

Supplementary Table 4: Comparison of the SNPs included in PRS calculation from this study and other published studies. See Supplementary_Table_4
